# Phase II single vs hypofractionated irradiation for timely access to partial breast radiotherapy (SHIFT-PB)

**DOI:** 10.1186/s12885-025-14720-w

**Published:** 2025-08-08

**Authors:** Robert Olson, Marjorie Cua, Quinn Matthews, Jordanna Laing, Dylan Narinesingh, Alan Nichol, Tanya Berrang, Theodora Koulis, Tania Karan, Nick Chng

**Affiliations:** 1 University of British Columbia, Vancouver, Canada; 2 University of Northern British Columbia, Prince George, Canada; 3 BC Cancer—Prince George, 1215 Lethbridge Street, Prince George, BC V2M7A9 Canada; 4 BC Cancer—Surrey, Surrey, BC Canada; 5 BC Cancer—Vancouver, Vancouver, BC Canada; 6 BC Cancer—Victoria, Victoria, BC Canada; 7 Department of Radiation Oncology, BC Cancer—Centre for the North, 1215 Lethbridge Street, Prince George, BC V2M 7E9 Canada

**Keywords:** Early-stage breast cancer, Partial breast irradiation, Single fraction

## Abstract

**Background:**

Breast cancer is the leading cause of global cancer incidence. In early-stage disease, standard treatment with breast-conserving surgery followed by whole breast irradiation (WBI) is associated with excellent outcomes. Multiple studies with extensive follow-up periods have demonstrated the comparative efficacy and toxicity outcomes of partial breast irradiation (PBI) in contrast to WBI. Various dose fractionation schedules for PBI have been recommended in clinical practice guidelines. In British Columbia (BC), a dose of 26 Gy in 5 fractions has been adopted. Early studies on single-fraction (SF) radiation for PBI have investigated its safety and effect on cosmetic outcomes, with promising initial results. In the context of the ongoing health care crisis, single-fraction PBI has the potential to reduce wait times and improve access to radiation. This study will therefore investigate a single-fraction PBI dose of 13 Gy.

**Methods:**

This is a phase II randomized controlled trial, with a primary objective of testing the feasibility of randomizing participants to 1 vs. 5 fractions of PBI for early stage, node negative, breast cancer. The primary endpoint is the ability to accrue 60 participants at 4 of the 6 BC Cancer centres over a 2- year period and to randomize them to 1 vs. 5 fractions of radiotherapy for PBI. Its secondary endpoints are time from CT simulation to partial breast radiotherapy, local control rates, quality of life as measured by Prospective Outcomes and Support Initiative (POSI)-Breast, rates of provider-rated toxicities as measured by Common Terminology Criteria for Adverse Events (CTCAE), rates of participant-reported toxicities as measured by participant reported outcome version of CTCAE (PRO-CTCAE), overall survival, and progression-free survival.

**Discussion:**

One of the trial’s objectives is testing the feasibility of randomizing participants to single vs. multiple fractions for PBI. If successful, it will lead to a phase III non-inferiority trial with the potential to inform breast cancer treatment guidelines. Ultimately, if found to be non-inferior, a single-fraction PBI can reduce wait times, facilitate access to radiation, and improve patient convenience, particularly for those in rural and remote communities who must travel long distances to receive high-quality cancer care.

**Trial registration:**

Clinicaltrials.gov identifier: NCT06885671. Date of Registration: 14 March 2025.

## Background

Breast cancer is the leading cause of global cancer incidence, with an estimated 2.3 million new cases being diagnosed worldwide in 2020 [1]. In high income countries, screening strategies result in detection of early-stage breast cancers resulting in a 5- year survival rate beyond 85% [2].

Standard treatment for early-stage breast cancer is surgery, oftentimes followed by adjuvant treatment such as radiotherapy and hormonal therapy. Prior to advancements made in the 1970s, the surgical approach to treating breast cancer relied heavily on the radical Halsted mastectomy. However, subsequent research revealed that employing breast-conserving surgery alongside radiotherapy yielded comparable outcomes to the Halsted mastectomy for tumors measuring up to 5 cm in size [3–5]. Thus, breast-conserving surgery followed by whole breast irradiation (WBI) became standard of care treatment for early-stage breast carcinoma with excellent local recurrence rates and breast cancer-specific mortality [6, 7]. 

Over the past few decades, partial breast irradiation (PBI) has emerged as an alternative to WBI. PBI is a targeted radiation approach commonly administered post-lumpectomy, specifically targeting the tumor bed. This targeted therapy reduces the exposure to other nearby tissues such as lungs, heart, and chest wall. PBI is delivered through several different techniques, including brachytherapy, intraoperative therapy with electrons or photons, and External Beam Radiotherapy (EBRT) using a Linear Accelerator (LINAC) machine [8]. 

The comparative efficacy and toxicity outcomes of PBI in contrast to WBI have been documented across numerous randomized trials with extensive follow-up periods. The 2017 IMPORT-LOW trial recruited 2018 early-stage breast cancer participants and split them between standard WBI with 40 Gy in 15 fractions, reduced dose WBI with 36 Gy in 15 fractions, and PBI with 40 Gy in 15 fractions. Five-year results confirmed that local relapse was minimal across all trial groups and that non-inferiority was shown for both partial-breast and reduced-dose radiotherapy [9]. In another trial comparing WBI to PBI done in Denmark, 865 participants were also treated with 40 Gy over 15 fraction doses. 10-year follow-up results confirmed that PBI for early breast cancer does not increase the risk of breast morbidity and recurrence [10]. The 2019 RAPID non-inferiority trial further enrolled 2135 participants and randomized them to either WBI or PBI. PBI was found to be non-inferior to WBI in preventing ipsilateral breast tumor recurrence (IBTR) in women [11]. These large, randomized control trials have validated the safety and efficacy of PBI, while updating the ASTRO guidelines to advise PBI for early breast cancer participants post-breast-conserving surgery (BCS) [8]. 

The initial conventional accepted radiation dose for WBI was 50 Gy in 25 fractions. In the early 2000s, the START-B trial showed that hypo fractionated WBI with 40 Gy in 15 fractions was not inferior to the conventional 50 Gy in 25 fractions, and guidelines were updated to include both [12]. The FAST-Forward Trial challenged this standard, by comparing the standard dosage of 40 Gy in 15 fractions to 27 Gy in 5 fractions, and 26 Gy in five fractions. The results show that 26 Gy in five fractions over 1 week is non-inferior to the standard of 40 Gy in 15 fractions over 3 weeks for local tumour control and is as safe in terms of normal tissue effects up to 5 years [13]. This has now become a standard dosage for practicing radiation oncologists [8]. 

Initial studies on single-fraction (SF) radiation for PBI have investigated its safety and effect on cosmetic outcomes. A phase I/II single arm prospective clinical trial has confirmed that a SF dose of 20 Gy of radiation delivered 2 to 8 weeks after lumpectomy is a well-tolerated therapy that does not adversely impact cosmesis or quality of life as reported by physicians and participants [14]. Another phase I trial has demonstrated that SF PBI can be employed to safely increase the dose up to 30 Gy in a SF with low toxicity and without detriment to baseline cosmesis [15]. Although longer follow-up would be needed to confirm efficacy results, initial tumor control data from these studies are promising.

Like other medical services in Canada impacted by the ongoing health care crisis, cancer radiation treatment wait-lists are continually growing. This presents a systemic need for efficient and accessible treatments. Employing a single fraction for PBI is one promising radiation technique that could reduce wait times and improve access to radiation. Participants with early stage, node negative breast cancer will benefit from a shorter course of radiotherapy that will save them time and money. The regimen will also be particularly advantageous for participants from rural and remote communities, who must travel long distances to receive high quality cancer care. This phase II trial of SF PBI vs. multiple fraction PBI is therefore proposed.

The standard arm will use a dose of 26 Gy in 5 fractions, which has been adopted in the province for PBI. This dose fractionation is based on data from the FAST-Forward trial and extrapolation of its results alongside the findings of IMPORT-Low, a partial breast radiation trial that used 40 Gy in 15 fractions [9, 13, 16]. 

In the experimental arm, this study will investigate a single-fraction PBI dose of 13 Gy. A similar single-fraction dose has been used in CT-guided high-dose-rate brachytherapy for breast intraoperative radiation therapy. A phase I trial by Showalter et al. [17] and its subsequent phase II trial [18] both utilized 12.5 Gy, a dose that was found to be feasible and safe.

A meta-analysis of the START pilot and START-A trials, which investigated hypofractionation in breast radiation, estimated an α/β value of 3.5 Gy for locoregional relapse [19]. Findings from the FAST-Forward trial showed a similar α/β value for tumor control, at 3.7 Gy [13]. Using the linear quadratic model, a dose of 26 Gy in 5 fractions is equivalent to 41.13 Gy in 2-Gy Fractions (EQD2). A single fraction of 13 Gy is estimated to be iso-effective to the control dose, with an EQD2 of 39 Gy.

According to FAST-Forward data, α/β values for late normal tissue effects may be closer to 2 Gy than the 3 Gy estimated in previous studies [13]. Using an α/β value of 2, a course of 26 Gy in 5 fractions and a single-fraction of 13 Gy are estimated to have iso-effective EQD2 values of 46.8 Gy and 48.75, respectively.

## Methods

The objective of this phase II randomized controlled trial is to test the feasibility of randomizing participants to 1 vs. 5 fractions of partial breast irradiation (PBI) for early stage, node negative, breast cancer. It also aims to collect data on the time from CT simulation to partial breast radiotherapy, and to compare toxicity, local control, quality of life (QoL) as measured by POSI-Breast, health provider rated toxicity as measured by Common Terminology Criteria for Adverse Events (CTCAE), participant self-reported toxicity as measured by participant reported outcome version of CTCAE (PRO-CTCAE), overall survival (OS), and progression free survival (PFS), between the two arms.

### Primary endpoint

The primary endpoint of the study is the ability to accrue 60 participants at 4 of the 6 BC Cancer centres over a 2- year period and to randomize them to 1 vs. 5 fractions of radiotherapy for PBI. This will be measured by the number of participants accrued in each of the BC cancer centres over a 2-year period for randomization to 1 vs. 5 fractions of radiotherapy for PBI.

### Secondary Endpoint(s)

The secondary endpoints of the study are the following:

Time from CT simulation to partial breast radiotherapy.

- Measured as the time from the day of CT simulation to the first fraction of PBI for Arm 1 or day of single-fraction PBI for Arm 2.

Local control rates.

- Local control measured as the absence of ipsilateral in-breast recurrence, defined as histologic evidence of invasive or in situ breast cancer in the ipsilateral breast.

Quality of life.

- Assessed using the Prospective Outcomes and Support Initiative (POSI) for Breast Data questionnaire, administered at baseline, then at 2 weeks, 2 months, 6 months, 12, months, 18 months, and 24 months post-treatment.

Rates of provider-rated toxicities.

- Occurrences of adverse events as measured by CTCAE assessed during treatment then at 2 weeks, 2 months, 6 months, 12, months, 18 months, and 24 months post-treatment.

Rates of participant-reported toxicities.

- Occurrences of adverse events as measured by PRO-CTCAE assessed during treatment then at 2 weeks, 2 months, 6 months, 12, months, 18 months, and 24 months post-treatment.

Overall Survival (OS).

- Time from randomization to death from any cause, or last follow-up, whichever occurs first. [Time Frame: approximately at the end of year 2 (study completion)]

Progression-free survival (PFS).

- Time from randomization to disease progression at any site, death, or last follow-up, whichever occurs first. [Time Frame: at 6 weeks, 6 months, 12 months, 18 months, and approximately at the end of 24 months]

## Study design

**Fig. 1:**
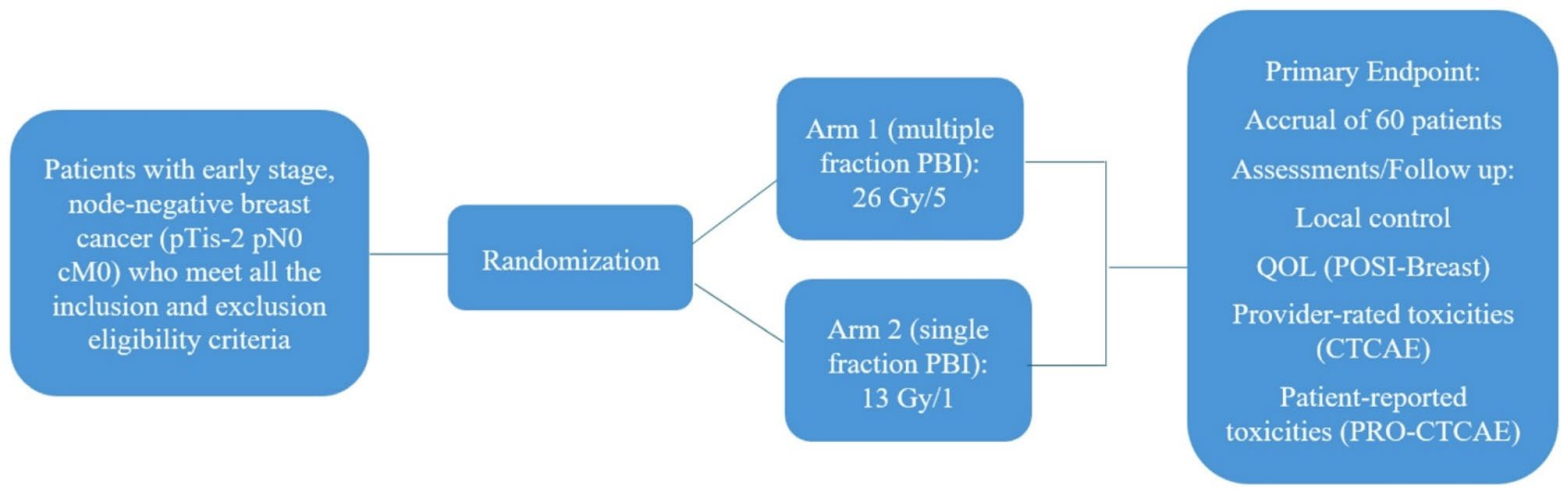
Trial Schema

This is a phase II open-label randomized controlled trial using a 1:1 randomization between two arms (26 Gy in 5 fractions vs. 13 Gy in 1 fraction PBI). Participating institutions will include the six BC Cancer sites. The trial schema is shown in Fig. 1.

## Inclusion criteria

- Female participants age 40 or older.
- Able to provide informed consent.
- pTis-2 pN0 cM0 breast cancer, with tumor size < 3 cm as per provincial guidelines.
- Able to complete electronic or paper entry of participant reported outcomes independently or with assistance from caregiver/family/friend/research staff.
- Eastern Cooperative Oncology Group (ECOG) performance status 0–2.
- A history and physical examination, including ECOG performance status performed within 8 weeks prior to trial enrollment.
- Participant is judged able to:
- Maintain a stable position during therapy.
- Tolerate immobilization device(s) that may be required to deliver PBI safely.
- Negative pregnancy test for People of Child-Bearing Potential (POCBP) *within 4 weeks of RT start date*.

## Exclusion criteria

### Participants are excluded from the study if any of the following criteria apply

- History of non-breast malignancies except adequately treated non-melanoma skin cancers, in situ cancers treated by local excision or other cancers curatively treated with no evidence of disease for ≥ 5 years.
- Uncontrolled concurrent malignant cancer.
- Seroma not visible.
- Ipsilateral implanted cardiac device.
- Prior radiotherapy requiring summation for planning.
- Inability to meet mandatory planning constraints.
- Requirement for a radiation boost (as determined by treating investigator).
- Positive lymph nodes.
- Positive surgical margins defined as “ink on tumor” (any invasive or DCIS cells on ink).
- Surgical cavities lacking clear delineation (surgical clips are not required but may assist in target delineation).
- Known germline BRCA1/2 mutation.
- Serious medical comorbidities precluding radiotherapy (i.e., connective tissue disorders such as lupus or scleroderma).
- Pregnant or breastfeeding.

## Pre-treatment evaluation

- History and Physical Examination within eight weeks of study registration (No exceptions to timelines):
- Including prior cancer therapies, and cancer-specific concomitant medications.
- Chemotherapy and surgery within twelve weeks of RT start date (No exceptions to timeline).
- Pregnancy test for people of child-bearing potential within 4 weeks of RT start date (No exceptions to timeline).

## Interventions

*Standard Arm—Multiple Fraction PBI (Arm 1)*.

Participants on Arm 1 will be treated with PBI with a dose of 26 Gy in 5 daily fractions.

*Experimental Arm—Single Fraction PBI (Arm 2)*.

Participants on Arm 2 will be treated with PBI with a dose of 13 Gy in 1 fraction.

### Immobilization

Immobilization and positioning must follow institutional guidelines. Use of angled versus flat breast boards must follow departmental practice; while flat positioning may be preferred for Volumetric Modulated Arc Therapy (VMAT) clearance, either approach is acceptable based on clinical and technical considerations. The participant will be positioned supine, with both arms (or only ipsilateral arm) over the head preferred, but positioning with both arms down is acceptable if needed. Non-supine positioning is permitted if clinically required, though expected to be uncommon.

### Imaging/Localization/Registration

All participants on both treatment arms will undergo CT simulation per the institution’s guidelines. Radio-opaque markers will be placed on the surgical scar. Markers may also be placed at the infraclavicular head and 2 cm inferior to the breast tissue and/or to delineate breast tissue. Wire localization may be used based on institutional preference but is not required. Other markers such as tattoos may also be used for daily localization and setup according to institutional guidelines. Axial CT images will be obtained, with the scan range according to institutional guidelines.

Deep inspiration breath hold (DIBH) may be used as per institutional guidelines.

Image guidance is required for treatment delivery, with imaging using either kV or cone-beam computed tomography (CBCT) permitted, depending on departmental practices.

For image guidance, relevant clips that are within or adjacent to the lumpectomy are contoured. Image-guided radiation therapy (IGRT) will be based on a clip match.

In the absence of clips, the seroma or an alternate surrogate (e.g., chestwall, scar wire) visible on kV or CBCT should be used.

For patients treated in the single-fraction arm, intrafraction imaging is suggested to verify or adjust patient positioning to correct for the increased risk of intrafraction motion.

All patients will have a day 1 post-treatment CBCT performed with the treated volume visible in the CBCT field of view, if technically feasible without any collisions. This CBCT will be saved within the ARIA system for retrospective analysis. CBCT acquisition parameters as per local departmental practice.

### Volume definitions

1. *Clinical Target Volume (CTV)*: Lumpectomy (as defined on CT simulation with the aid of surgical clips, if present) + 1.0–1.5 cm 3D expansion. The CTV will be limited posteriorly by the pectoral fascia and anteriorly by the skin surface, cropped to anatomical boundaries (lung, chest wall, ribs, sternum, muscles, etc.) and 0.5 cm below the skin surface per RO discretion.
2. *Planning Target Volume (PTV)*: CTV + 0.7–1.0 cm 3D expansion (recommended margins may vary depending on physics guidelines considering setup, immobilization, and IGRT practices). 1.0 cm is recommended, particularly for DIBH, large or mobile breast, or lumpectomy > 15 cc (margin may be reduced in consultation with physics).
3. *PTV_Eval*: PTV cropped back to 0.5 cm below the skin surface, and 0.5 cm from lung, but including any CTV within 0.5 cm of lung.

### Organs at risk (OAR) doses

Delineation of organs at risk (OARs) is required and must include the ipsilateral and contralateral lung, heart, and ipsilateral and contralateral breast. Constraints for OARs for the five-fraction regimen are based on provincial practice and protocols from other studies using a similar number of fractions such as the FAST-Forward and the Florence PBI trials [13, 20]. Constraints for single-fraction radiation were determined by calculating the corresponding EQD2 from the five-fraction regimen using an α/β value of 2, and then rounded or adjusted for conservativeness based on trial group consensus opinion. OAR dose constraints are listed in Table 1.

The “Ipsilateral breast-PTV” constraint is applied to the ipsilateral breast structure with the PTV subtracted. All OAR constraints are prioritized above PTV coverage and homogeneity metrics, except for contralateral breast D0.035 cc which is the lowest priority and should be exceeded if necessary to achieve all other constraints. All planning constraints (except contralateral breast) are mandatory trial requirements.

**Table 1:** SHIFT-PB planning constraints

Mandatory Planning Constraints				
OAR	Fractions			
	5		1	
Ipsilateral lung	V10 Gy	≤ 20%	V5 Gy	≤ 20%
Heart	V7 Gy	≤ 5%	V4 Gy	≤ 5%
	V3 Gy	≤ 10%	V2 Gy	≤ 10%
	V1.5 Gy	≤ 30%	V1 Gy	≤ 30%
Contralateral lung	V5 Gy	≤ 10%	V3 Gy	≤ 10%
Ipsilateral breast-PTV	V50%	≤ 50 [60]%	V50%	≤ 50 [60]%
Target Planning Goals (both arms)				
PTV_eval	V95%		≥ 98 [95]%	
	V105%		≤ 5 [10]%	
Body	D0.035 cc		≤ 107 [110]%	
Non-Mandatory OAR Constraints (both arms)				
Contralateral breast	D0.035 cc		≤ 1 [2] Gy	

Acceptable values indicated in brackets where applicable

### Treatment planning

Planning must be performed using VMAT with a minimum of two partial arcs, as appropriate, to minimize entrance dose through contralateral tissues. The selection of beam energy is at the discretion of the treating department. Optimization must be performed to the fully expanded PTV (or surrogate opti structure) to ensure robust target coverage during treatment considering setup uncertainty and intrafraction motion. Use of temporary planning structures to ensure sufficient build-up to the fully expanded PTV for optimization is mandatory, though the exact method is not prescribed. Flash buildup as per departmental practice– with a recommended thickness of 0.5–1.0 cm to the PTV and a HU of -500– is an example. After optimization, these structures should be removed, and the dose recalculated without buildup, then evaluated on the PTV_eval.

### Target planning goals—both arms

The volume of the PTV_eval receiving 95% of the prescription dose should be ≥ 98% (preferred), or ≥ 95% (mandatory).

The maximum dose to 0.035 cc of any tissue should be ≤ 107% (preferred), or ≤ 110% (mandatory) of the prescription dose. Maximum dose should fall within the PTV.

The volume of the PTV_eval receiving ≥ 105% of the prescription dose should be ≤ 5% (preferred) or ≤ 10% (mandatory).

### Quality assurance (QA) (Arms 1 & 2)

In order to ensure participant safety and effective treatment delivery, a robust quality assurance protocol is incorporated. The following requirements must be completed for each participant:

- Prior to treatment, each participant must be peer reviewed by a radiation oncologist with PBI expertise. Peer review of contours must occur before treatment. QA rounds are optional.
- All treatment plans must undergo physics QA checks per institutional practice.
- All PBI treatments must be delivered on a radiotherapy linac appropriate for VMAT PBI and compliant with a QA schedule as recommended by current international published guidelines. *As this study will only involve BC Cancer sites, formal credentialling of centres will not be required, as it is assumed that all treatments will be delivered on RT linacs appropriate for VMAT PBI and with QA schedule as recommended by guidelines.

Peer review of contours must be done within a treating centre and must follow institutional guidelines. As this is a BC Cancer-only trial, acceptable documentation includes use of the provincial ARIA platform. The completed peer review form can be printed and uploaded to REDCap for trial tracking purposes.

### Systemic therapy

Hormone therapy and targeted cancer drugs, such as trastuzumab (Herceptin), are permitted. The use of systemic therapy that are cytotoxic, immunotherapeutic, or molecularly targeted agents are not allowed within 2 weeks prior to radiation until 1 week after the last fraction. Investigational therapy is prohibited within 14 days prior to initiation of study treatment and during study treatment.

### Data handling and recordkeeping

All participant data for the study for the study must be recorded on printed or electronic case report forms (CRF), unless electronically transmitted to the sponsor or designee, with the investigator responsible for verifying accuracy by signing the CRF. Monitoring details describing strategy, methods, responsibilities and requirements, including handling of noncompliance issues and monitoring techniques (central, remote, or on-site monitoring) are provided in the SHIFT-PB Monitoring Plan. Study monitors will verify source data to ensure accuracy, participant safety, and compliance with protocols, International Council for Harmonization (ICH) Good Clinical Practice (GCP), and regulations.

### Patient discontinuation / withdrawal

A participant may voluntarily withdraw from the study at any time at his/her own request. Participants may also be withdrawn at any time at the discretion of the investigator if any exclusion/inclusion criteria have been violated during the study, they are unable to meet the mandatory planning requirements after enrollment, or for safety reasons, etc.

At the time of discontinuing from the study, if possible, an early discontinuation visit must be conducted. The clinical evaluations that would have been performed at the end of the study should be obtained. If a participant is removed because of an adverse or serious event, they should remain under medical observation as long as deemed appropriate by the treating physician and follow recording and reporting requirements.

Participants withdrawn or discontinued can be replaced at the discretion of the sponsor-investigator/Study Principal Investigator.

### Follow-up evaluation and assessment of efficacy

After treatment, participants will be followed up after 2 weeks. Clinical follow-up appointments will then be scheduled at 2 months, 6 months, 12 months, 18 months, and 24 months, and may be conducted via telephone or videolink. Follow-up visits requiring a physical exam may still be conducted via telephone/videolink if the physical exam is performed within the specified window by the participant’s Family Practitioner (FP) or General Practitioner in Oncology (GPO), with documentation. Otherwise, the participant must return to the clinic for the physical exam to be performed on-site. At each visit, a medical history will be conducted by the oncologist, and CTCAE toxicities recorded using the adverse events form, as well as ECOG scores. The POSI-Breast questionnaire is to be completed at baseline and on each follow-up visit.

A mammogram will be done beginning 6 months after treatment, then annually thereafter. Cytological or histological confirmation of the ipsilateral and contralateral primary tumor is required, including at the time of recurrence.

There is no planned follow-up at the end of the study. Additional care or follow-up assessments that will be provided to participants after they complete or discontinue the study will involve standard of care treatment for what is normally expected for their condition.

## Statistical considerations

### Randomization

This study will employ a 1:1 randomization between Arm 1 and Arm 2, with further stratification by centre. Permuted block randomization will be used to reduce selection bias, promote allocation concealment, and improve balance across groups over the trial period. Participants will be randomized based on computer-generated random permuted blocks using a block size based on a multiple of 2 (with the block size known only to statistician until analysis is completed). This is an open-label randomized controlled study design, however, outcome assessors and data analysts will be blinded to the identity of each treatment arm.

The Coordinating Centre will be responsible for randomization of participants using a randomization module in the REDCap Electronic Data Capture (EDC) system, which will be used for trial data collection. In conjunction with trial statistician and Site Principal Investigators, the Coordinating Centre will be responsible for storing and analyzing trial data.

### Number of participants/sample size

The study aims to enroll 60 participants in at least 4 out of 6 BC cancer centres. The sample size of 60 participants was selected based on feasibility across multiple sites and to allow for meaningful descriptive analyses.

### Statistical methods

Descriptive statistics will be used to report the number of participants accrued, descriptions of participant characteristics, time interval from CT simulation to plan approval, POSI scores, and rates of local control and adverse events.

PFS and OS will be calculated using the Kaplan-Meier method with differences compared using the stratified log-rank test.

### Data safety monitoring committee (DSMC)

The DSMC will be made up of the study co-investigators and will review data relating to safety and efficacy to ensure the continued scientific validity and merit of the study. If the DSMC deems that toxicity rates are excessive (> 25% grade 3, or > 5% grade 4 or 5 toxicity), then the DSMC can, at its discretion, recommend cessation of the trial, dose adjustment, or exclusion of certain subsets of the study population that are deemed as high-risk for complications.

## Ethical considerations

The Principal Investigator will obtain ethical approval and clinical trial authorization by competent authorities according to local laws and regulations.

### Institutional review board (IRB) / research ethics board (REB)

The protocol (and any amendments), the informed consent form, and any other written information to be given to subjects will be reviewed and approved by a properly constituted Institutional Review Board (IRB)/Research Ethics Board (REB), operating in accordance with the current federal regulations International Council for Harmonization (ICH) Good Clinical Practice (GCP) and local regulatory requirements. A letter to the investigator documenting the date of the approval of the protocol and informed consent form will be obtained from the IRB/REB prior to initiating the study. Any institution opening this study will obtain IRB/REB approval prior to local initiation and will be responsible for maintaining approval throughout the duration of the trial. Principal Investigators must provide evidence of IRB/REB approval on an annual basis.

### Informed consent

The written informed consent form is to be provided to potential study patients should be approved by the IRB/ REB and adhere to ICH GCP and the ethical principles that have their origin in the Declaration of Helsinki. The investigator is responsible for obtaining written informed consent from each patient, or if the patient is unable to provide informed consent, the patient’s legally acceptable representative, prior to beginning any study procedures and treatment(s). The investigator should inform the patient, or the patient’s legally acceptable representative, of all aspects of the study, including the potential risks and benefits involved. The patient should be given ample time and opportunity to ask questions prior to deciding about participating in the study and be informed that participation in the study is voluntary and that they are completely free to refuse to enter the study or to withdraw from it at any time, for any reason. The informed consent must be signed and dated by the patient, or the patient’s legally acceptable representative, and by the person who conducted the informed consent discussion. A copy of the signed and dated written informed consent form should be given to the patient or the patient’s legally acceptable representative. The process of obtaining informed consent should be documented in the patient source documents.

### Confidentiality of subject records

The names and personal information of study participants will be held in strict confidence. All study records (case report forms, safety reports, correspondence, etc.) will only identify the subject by initials and the assigned study identification number. The investigator will maintain a confidential subject identification list (Master List) during the course of the study. Access to confidential information (i.e., source documents and patient records) is only permitted for direct subject management and for those involved in monitoring the conduct of the study (i.e., Sponsors and their representatives, representatives of the IRB/REB, and regulatory agencies). The subject’s name will not be used in any public report of the study.

### Protocol amendments and trial publication

Any modifications to the trial protocol must be approved and enacted by the Principal Investigator. Protocol amendments will be communicated to all participating centres, investigators, IRBs, and trial registries by the principal investigator. Any communication or publication of trial results will be led by the principal investigator and is expected to occur within 1 year of the primary analysis. Trial results will remain embargoed until conference presentation of an abstract or until information release is authorized. Authorship of the trial abstract and ultimately the full manuscript will be decided by the principal investigator at the time of submission. Professional writers will not be used for either abstract or manuscript preparation.

### Trial management

The Coordinating Centre for this study will be responsible for trial activities at all participating sites. The Coordinating Centre will be responsible for randomization of participants. In conjunction with trial statistician and Site Principal Investigators, the Coordinating Centre will be responsible for storing and analyzing trial data. Designated Site Principal Investigators will be responsible for reviewing eligibility of participants at their centre.

## Discussion

Breast cancer remains the leading cause of global cancer incidence [1]. For early breast cancer, standard of care with breast-conserving surgery followed by whole breast irradiation is associated with excellent outcomes in terms of local recurrence rates and breast cancer-specific mortality. Multiple studies with extensive follow-up periods have documented the comparative efficacy and toxicity outcomes of PBI in contrast to WBI [9–11]. Various dose fractionation schedules for PBI have been recommended in the ASTRO clinical practice guideline [8]. In British Columbia, a dose of 26 Gy in 5 fractions has been adopted for PBI based on data from the FAST-Forward trial and extrapolation of its results alongside the findings of IMPORT-Low [9, 13, 16]. 

This phase II trial will investigate a single-fraction PBI dose of 13 Gy. This experimental dose was determined to be iso-effective to the control dose of 26 Gy in 5 fractions for tumor control and late normal tissue toxicity, as estimated using the linear-quadratic model and supported by existing literature [13, 19]. 

One of the trial’s objectives is testing the feasibility of randomizing participants to single vs. multiple fractions. If successful for this outcome, a subsequent non-inferiority phase III randomized controlled trial will be undertaken. This has a potential to inform practice guidelines in breast cancer treatment.

Ultimately, if found to be non-inferior, a single-fraction PBI can reduce wait times, facilitate access to radiation, and improve patient convenience, particularly for those in rural and remote communities who must travel long distances to receive high-quality cancer care.

## Data Availability

No datasets were generated or analysed during the current study.

## Abbreviations

AE: Adverse event
ASTRO: American society for radiation oncology
BCS: Breast-conserving surgery
CBCT: Cone-beam computed tomography
CRF: Case report form
CT: Computedtomography
CTCAE: Common terminology criteria for adverse events
CTV: Clinical rarget volume
DIBH: Deep inspiration breath hold
DSMC: Data safety monitoring committee
EBRT: External beam radiotherapy
ECOG: Eastern cooperative oncology group
FP: Family practitioner
GCP: Good clinical practice
GPO: General practitioner in oncology
IBTR: Ipsilateral breast tumor recurrence
ICF: Informed consent form
ICH: International council for harmonization
LINAC: Linear accelerator
OAR: Organ at risk
OS: Overall survival
PBI: Partial breast irradiation
PFS: Progression free survival
POCBP: People of child-bearing potential
POSI: Prospective outcomes and support initiative
PRO: Patient reported outcomes
PTV: Planning target volume
QoL: Quality of life
REB: Research ethics board
RT: Radiation therapy
SAE: Serious adverse event
SF: Single fraction
VMAT: Volumetric modulated arc therapy
WBI: Whole breast irradiation
